# Supplementary material for: Dynamic nucleolar phase separation influenced by non-canonical function of LIN28A instructs pluripotent stem cell fate decisions
Source: Nat Commun. 2024 Feb 10;15:1256. doi: 10.1038/s41467-024-45451-4 (PMC10858886; doi:10.1038/s41467-024-45451-4)
Supplement: Supplementary file 1 — Supplementary Information [file 41467_2024_45451_MOESM1_ESM.pdf]

Supplementary Figure 1

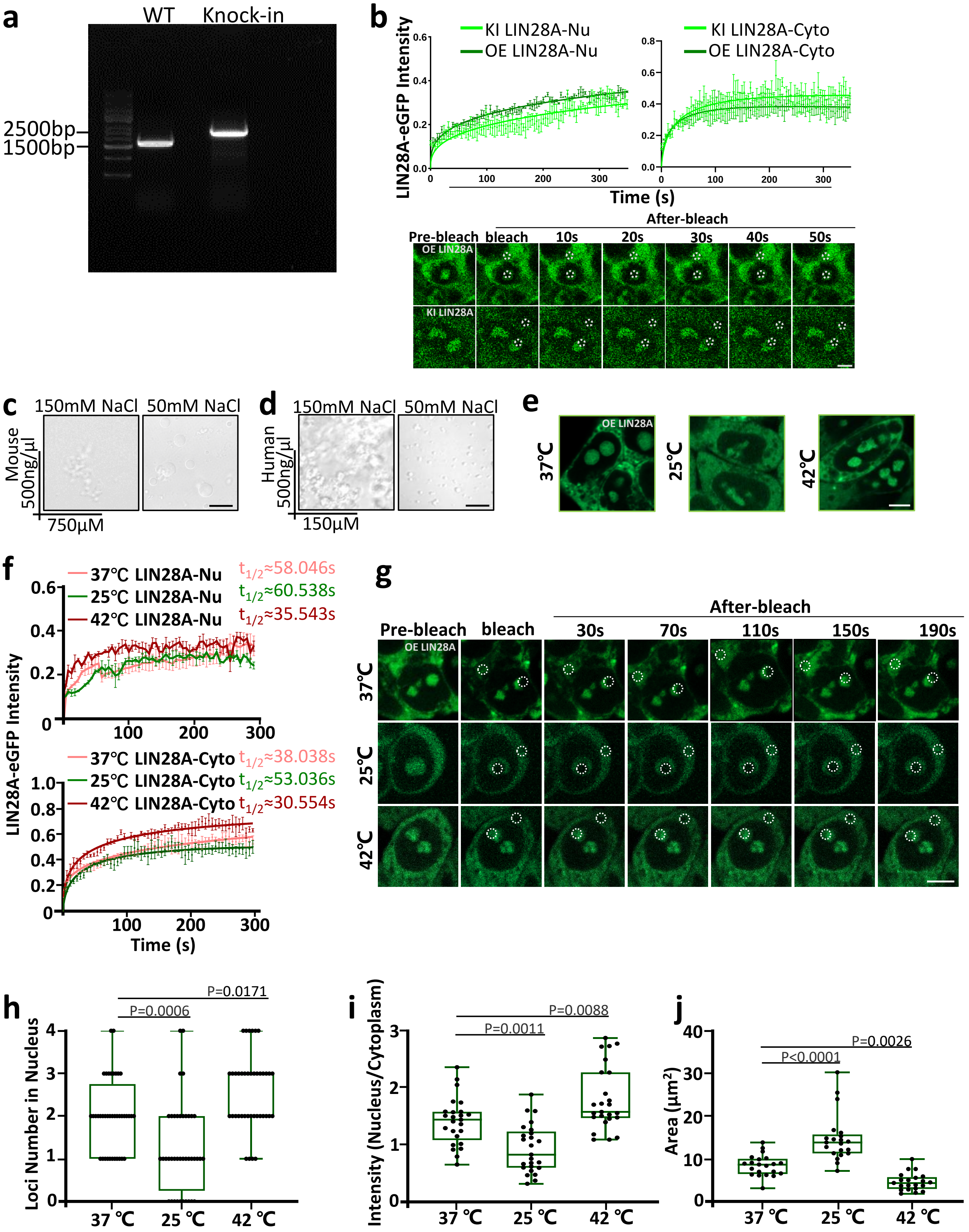

Supplementary Figure 1 Over-expressed(OE) eGFP-LIN28A construct behavior compares to endogenous LIN28A localization/behavior, LIN28A protein showed temperature-sensitive phase separation in cells, and LIN28A phase separation in the presence of 50 mM and 150 mM NaCl *in vitro* .

a, PCR experiments showing that the knock-in mESCs genome contains the inserted 700 base fragment compared with wild-type mESCs. This result indicates that the eGFP was successfully knocked-in(homozygotes). **b,** FRAP analysis and images showing overexpressed eGFP-LIN28A and knock-in eGFP-LIN28A recovery after photobleaching in the nucleus. n = 3 biologically independent experiments. Data are presented as mean values +/− SEM. SEM: standard error of the mean. Scale bar, 5μm. **c,** Representative images of mouse LIN28A recombinant protein forms irregular aggregation or droplets in the presence of total RNA (extracted from ESCs) in vitro. Scale bar, 10μm. **d,** Representative images of human LIN28A recombinant protein forms irregular aggregation or droplets in the presence of total RNA (extracted from ESCs) in vitro. Scale bar, 10μm.

e, Confocal microscopy imaging of the live eGFP-LIN28A cells at 37℃, 25℃ and 42℃. Scale bar, 5μm. **f,** FRAP analysis showing temperature shock impacted recovery after photobleaching of eGFP-LIN28A; n= 3 biologically independent experiments. Data are presented as mean values +/− SEM. **g,** Representative confocal microscopy images of recovery after photobleaching (FRAP) of eGFP-LIN28A in living WT cells at three different temperatures. The targeted bleached region is highlighted in a white circle. Scale bar, 5μm. **h,** Statistical analysis of LIN28A protein loci numbers in the nucleus at three different temperatures (37℃, 42℃ and 25℃; n=40). One-way ANOVA. **i,** Statistical analysis of LIN28A nucleus/cytoplasm fluorescence intensity ratio at three different temperatures (37℃, 42℃ and 25℃; n=25). One-way ANOVA. **j,** Statistical analysis of LIN28A loci area in the nucleus at three different temperatures (37℃, 42℃ and 25℃; n=21). One-way ANOVA. For **h-j**, the center line is the median, the bottom of the box is the 25th percentile boundary, the top of the box is the 75th and the top and bottom of the vertical line define the boundary of the data. Source data are provided as a Source Data file.

Supplementary Figure 2

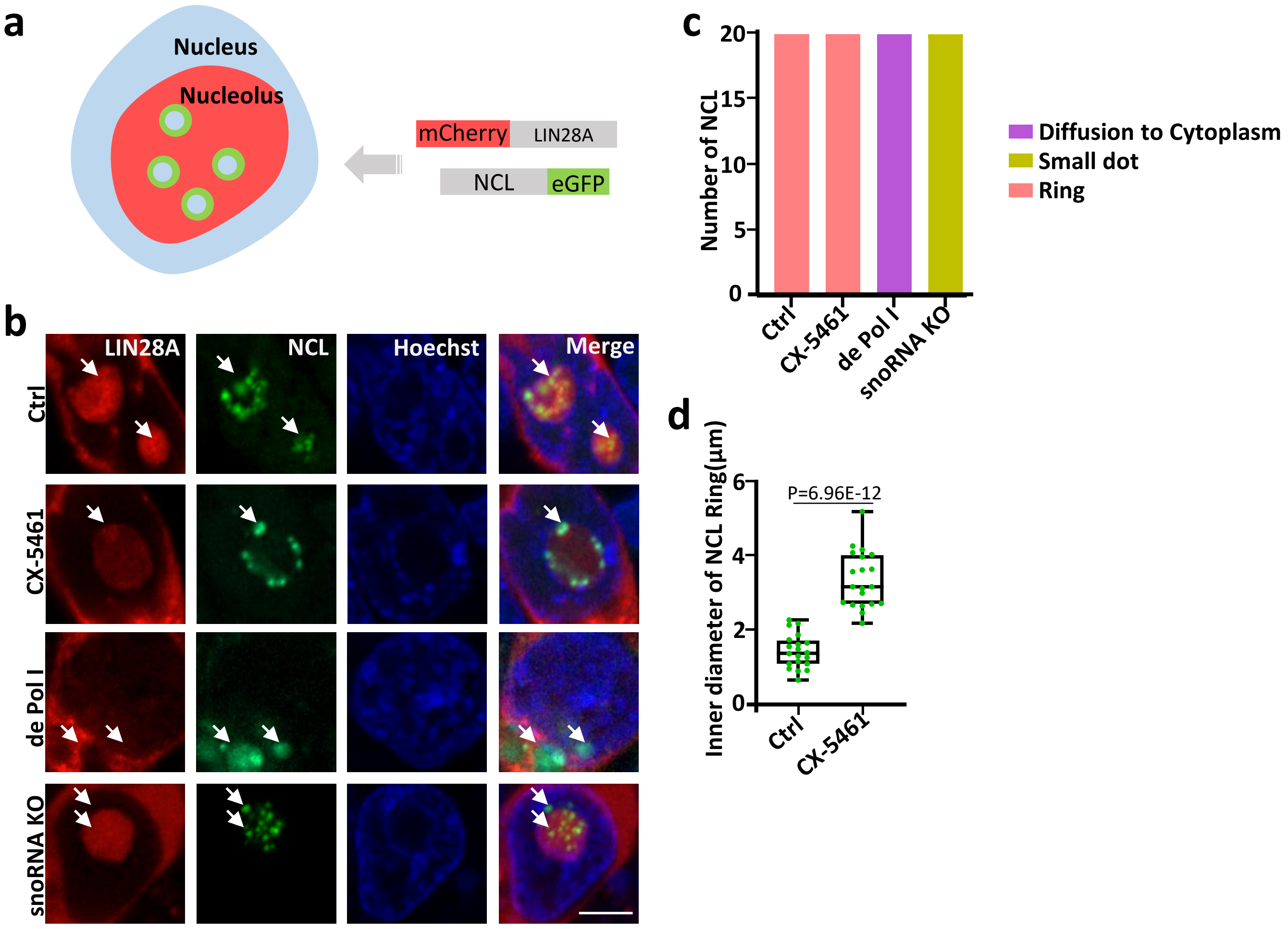

Supplementary Figure 2 The morphology and nucleolar localization of LIN28A and nucleolar protein NCL in three kinds of ESCs with defects in rRNA biogenesis.

a, Schematic representation of the mCherry-LIN28A/NCL- eGFP cell line generated. **b,** Representative images of the morphology and nucleolar localization of LIN28A and NCL in living ESCs of control cell, CX-5461 treatment cells, Pol I degraded cells, and snoRNA knockout cells. Scale bar, 5µm. n= 3 biologically independent experiments. **c,** Statistical analysis of the numbers the typical morphology of NCL in living ESCs of control cell, CX-5461 treatment cells, Pol I degraded cells, and snoRNA knockout cells; n=20 nucleoli. Fisher Exact Test,two-sided; Ctrl vs CX-5461:1, Ctrl vs de Pol I:p=1.45089E-11, Ctrl vs snoRNA KO:p=1.45089E-11. **d,** Statistical analysis of the inner diameter of NCL ring in living ESCs of control cell and CX-5461 treatment cells, n=20 nucleoli, two-tailed unpaired student's t-test. The center line is the median, the bottom of the box is the 25th percentile boundary, the top of the box is the 75th and the top and bottom of the vertical line define the boundary of the data. Source data are provided as a Source Data file.

Supplementary Figure 3

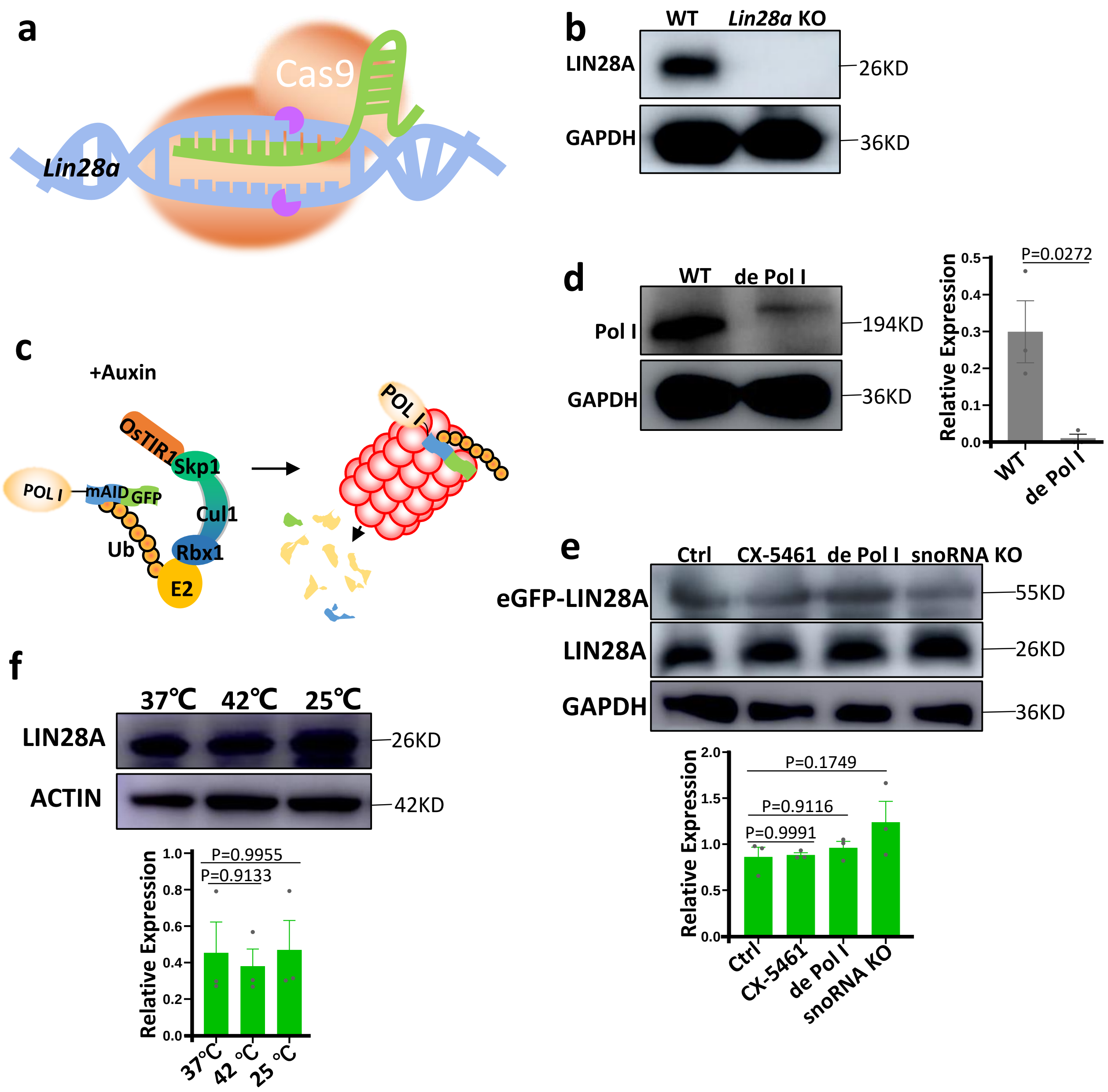

Supplementary Figure 3 The expression level of LIN28A proteins detected by immunoblotting experiments.

a, A cartoon illustrating CRISPR/CAS9 technology. **b,** Immunoblotting and statistical analysis showed LIN28A protein knockout with CRISPR/CAS9, n = 3 biologically independent experiments. **c,** A cartoon illustrating Pol I degradation system with the degron technology. **d,** Immunoblotting and statistical analysis showed the level of Pol I; normalized to GAPDH; n = 3 biologically independent experiments, two-tailed unpaired student's t-test. **e,** Immunoblotting and statistical analysis showed LIN28A expression level in control, CX-5461-treated, Pol I degraded and snoRNA knockout mESCs; normalized to GAPDH; n = 3 biologically independent experiments. One-way ANOVA. **f,** Immunoblotting and statistical analysis showed LIN28A expression level in mESCs at 37℃, 42℃, 25℃; normalized to GAPDH or ACTIN; n = 3 biologically independent experiments. One-way ANOVA. For **d-f**, data are presented as mean values +/− SEM. Source data are provided as a Source Data file.

Supplementary Figure 4

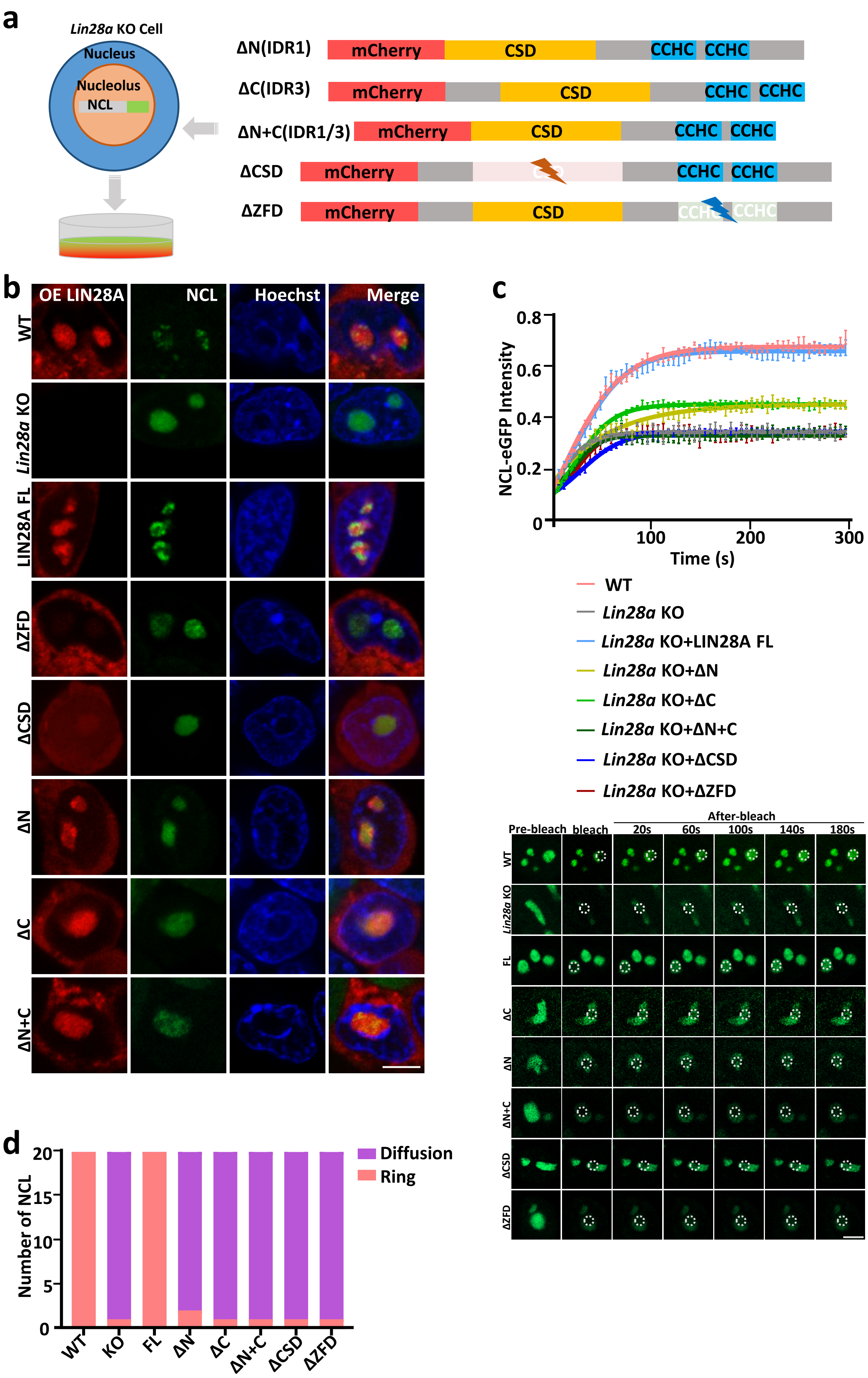

Supplementary Figure 4 Truncated LIN28A deformed the morphology and fluidity of nucleolar protein NCL. **a,** A schematic diagram of the constructs used to investigate the function of individual domains of LIN28A in NCL-eGFP stable expression *Lin28a* KO cells. **b,** Representative confocal microscopy images of the morphology and nucleolar localization of LIN28A and NCL in living WT, *Lin28a* KO, and *Lin28a* KO ESCs transduced with full length WT LIN28A or individual domain deleted LIN28A variants. Scale bar, 5μm. n = 3 biologically independent experiments. **c,** FRAP analysis showing NCL-eGFP recovery after photobleaching in the above cells in (b), and representative images of NCL fluorescence recovery after photobleaching (FRAP) in living WT, *Lin28a* KO, and *Lin28a* KO ESCs transduced with full length WT LIN28A or individual domain deleted LIN28A variants. Scale bar, 5μm. n=3 biologically independent experiments. Data are presented as mean values +/− SEM. **d,** Statistical analysis of the numbers the typical morphology of NCL in the WT, *Lin28a* KO, LIN28A full-length LIN28A overexpressing, and truncated LIN28A overexpressing cells; n=20 nucleoli. Fisher Exact Test, two-sided; WT vs KO:p=3.05E-10, WT vs FL:p=1, WT vs ΔN:p=3.35E-09, WT vs ΔC:p=3.05E-10, WT vs ΔN+C:p=3.05E-10, WT vs ΔCSD:p=3.05E-10, WT vs ΔZFD:p=3.05E-10. Source data are provided as a Source Data file.

Supplementary Figure 5

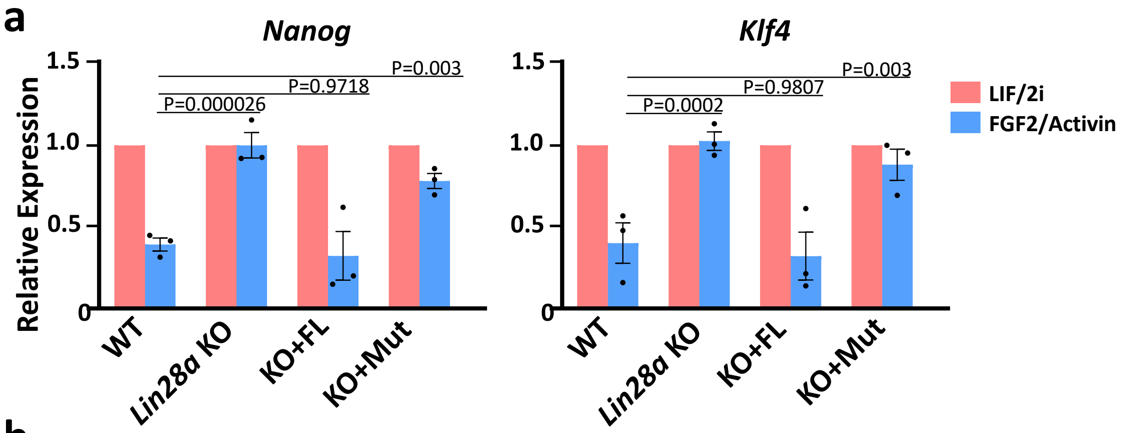

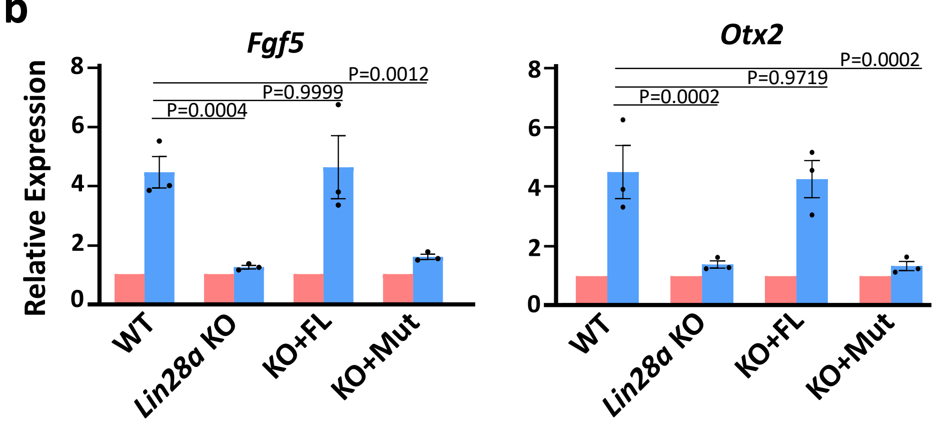

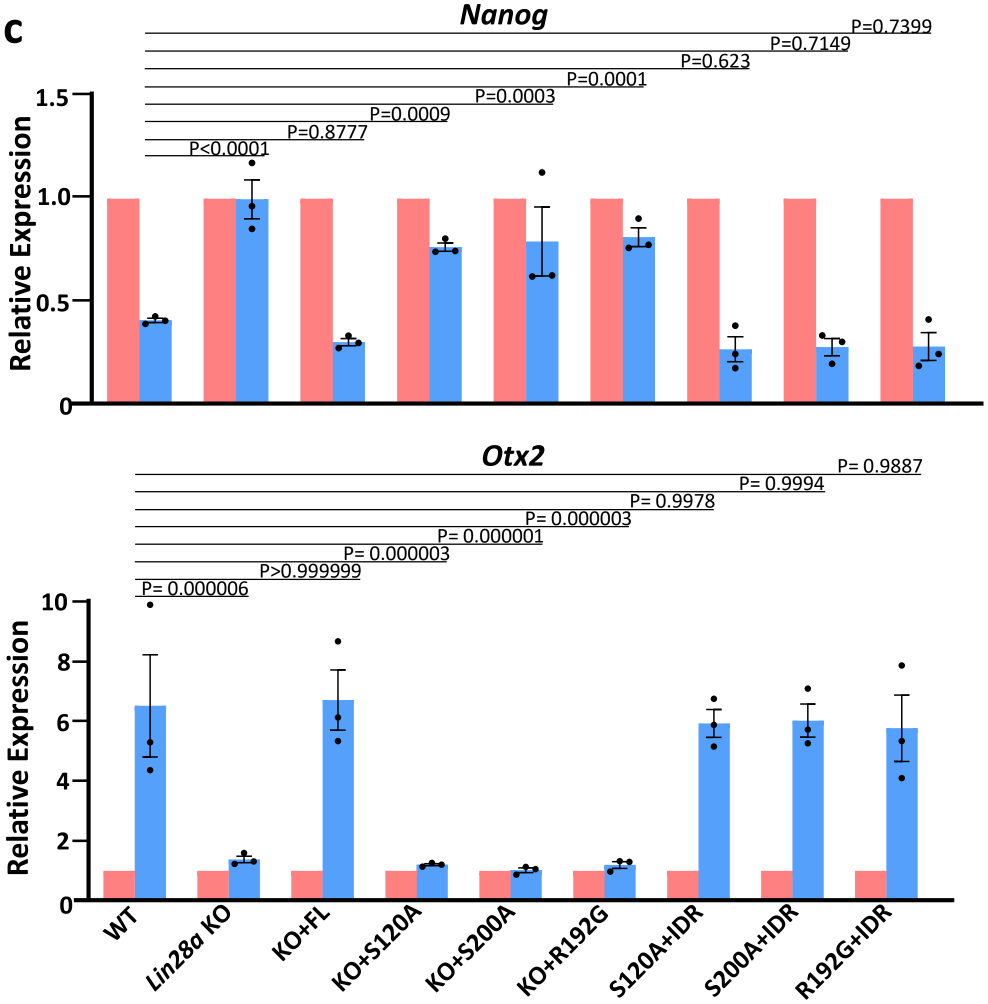

Supplementary Figure 5 qPCR analysis of the naïve and primed state genes indicated that normal LIN28A phase separation promoted primed pluripotency conversion.

a, qRT-PCR showing the naïve pluripotent marker gene expression in WT, *Lin28a* KO, and *Lin28a* KO ESCs transduced with full length WT LIN28A or Mut-LIN28A, cultured in the naïve and primed state conditions. n = 3 biologically independent experiments. Data are presented as mean values +/− SEM. Two-way ANOVA. **b,** qRT-PCR showing the primed pluripotent marker gene expression in WT, *Lin28a* KO, and *Lin28a* KO ESCs transduced with full length WT LIN28A or Mut-LIN28A, cultured in the naïve and primed state conditions. n = 3 biologically independent experiments. Data are presented as mean values +/− SEM. Two-way ANOVA. **c,** qRT-PCR showing the naïve and primed pluripotent marker gene expression in WT, *Lin28a* KO, and *Lin28a* KO ESCs transduced with full length WT LIN28A or the indicated LIN28A variants, cultured in the naïve and primed state conditions. n = 3 biologically independent experiments. Data are presented as mean values +/− SEM. Two-way ANOVA. Source data are provided as a Source Data file.

Supplementary Figure 6

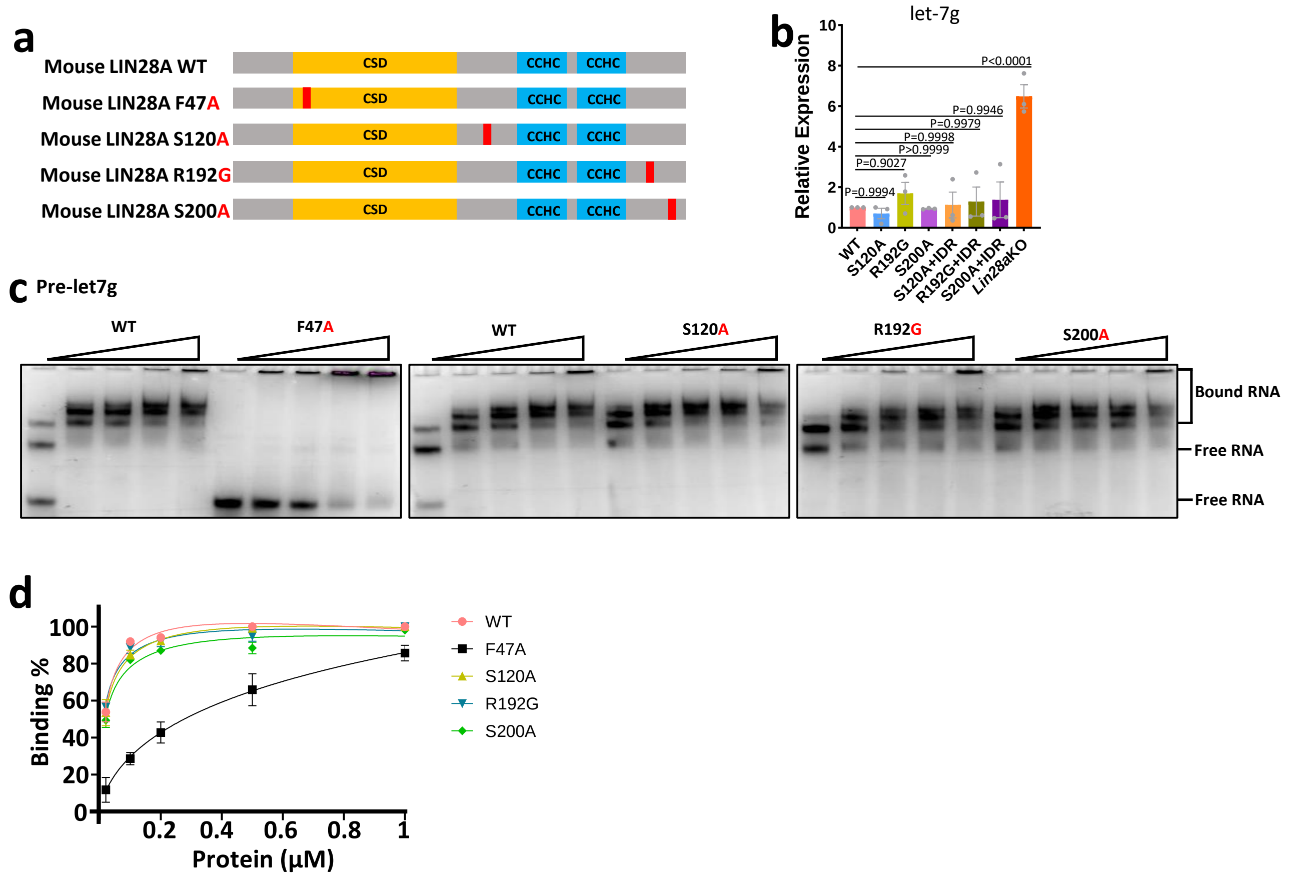

Supplementary Figure 6 Electrophoretic mobility shift assay (EMSA) and qPCR showing WT LIN28A's and LIN28A variants' affinity for pre-let-7g.

a, Constructs used to investigate the function of mutations of mouse LIN28A. **b,** qRT-PCR showing the let-7g expression in WT, *Lin28a* KO, and LIN28A mutations ESCs. n = 3 biologically independent experiments. Data are presented as mean values +/− SEM. Two-way ANOVA. **c,** EMSAs with Cy5-labeled pre-let-7g as probe, mixed with increasing concentrations (0.02, 0.1, 0.2, 0.5, and 1μM) of mouse LIN28A variants, n = 3 biologically independent experiments. **d,** Binding assays with Cy5-labeled pre-let-7g as probe, mixed with increasing concentrations of mouse WT LIN28A and LIN28A variants, n = 3 biologically independent experiments. Data are presented as mean values +/− SEM.

(0.02μM Protein, WT vs. F47A: P<0.0001, WT vs. S120A:P=0.9999, WT vs. R192G:P=0.9594, WT vs. S200A:P=0.7932.)
(0.1μM Protein, WT vs. F47A: P<0.0001, WT vs. S120A:P=0.4544, WT vs. R192G:P=0.9132, WT vs. S200A:P=0.1811.)
(0.2μM Protein, WT vs. F47A: P<0.0001, WT vs. S120A:P=0.9867, WT vs. R192G:P=0.9656, WT vs. S200A:P=0.4614.)
(0.5μM Protein, WT vs. F47A: P<0.0001, WT vs. S120A:P=0.9990, WT vs. R192G:P=0.7092, WT vs. S200A:P=0.0958.)
(1μM Protein, WT vs. F47A: P=0.0262, WT vs. S120A:P>0.9999, WT vs. R192G:P>0.9999, WT vs. S200A:P=0.9860.)
Source data are provided as a Source Data file.

Supplementary Figure 7

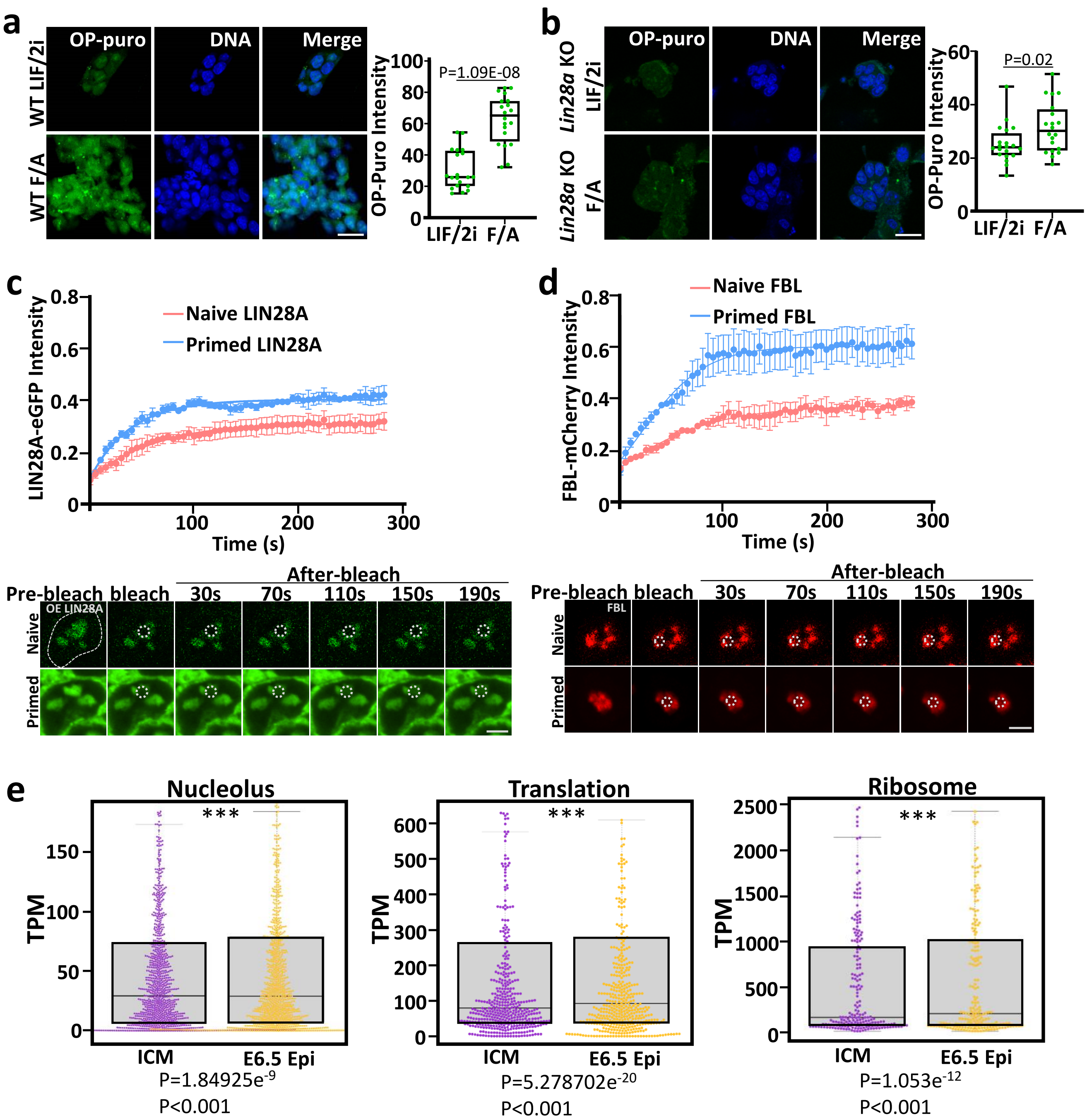

Supplementary Figure 7 Nucleolar become more active in primed pluripotency conversion.

a, Fluorescence imaging and OP-Puro intensity statistical analysis of OP-puromycin-labeled WT mESCs in naïve and primed state. n=20 cells. Scale bar, 100µm. Two-tailed unpaired student's t-test. **b,** Fluorescence imaging and OP-Puro intensity statistical analysis of OP-puromycin-labeled *Lin28a* KO mESCs in naïve and primed state. n=20 cells. Scale bar, 100µm. Two-tailed unpaired student's t-test. **c,** FRAP analysis showing LIN28A fluorescence recovery after photobleaching in the WT nucleoli in the naïve state and the primed state, and representative images of LIN28A fluorescence recovery after photobleaching (FRAP) in the WT nucleolus in the naïve state and the primed state. Scale bar, 5µm. n=3 biologically independent experiments. Data are presented as mean values +/− SEM. **d,** FRAP analysis showing FBL fluorescence recovery after photobleaching in the WT nucleoli in the naïve state and the primed state, and representative images of FBL fluorescence recovery after photobleaching (FRAP) in the WT nucleolus in the naïve state and the primed state. Scale bar, 5µm. n=3 biologically independent experiments. Data are presented as mean values +/− SEM. **e,** Boxplots demonstrating the expression levels of nucleolus, translation, and ribosome genes at ICM and E6.5 Epi. TPM, Transcripts Per Million mapped reads. For **a,b,e,** the center line is the median, the bottom of the box is the 25th percentile boundary, the top of the box is the 75th and the top and bottom of the vertical line define the boundary of the data. Source data are provided as a Source Data file.

Supplementary Figure 8

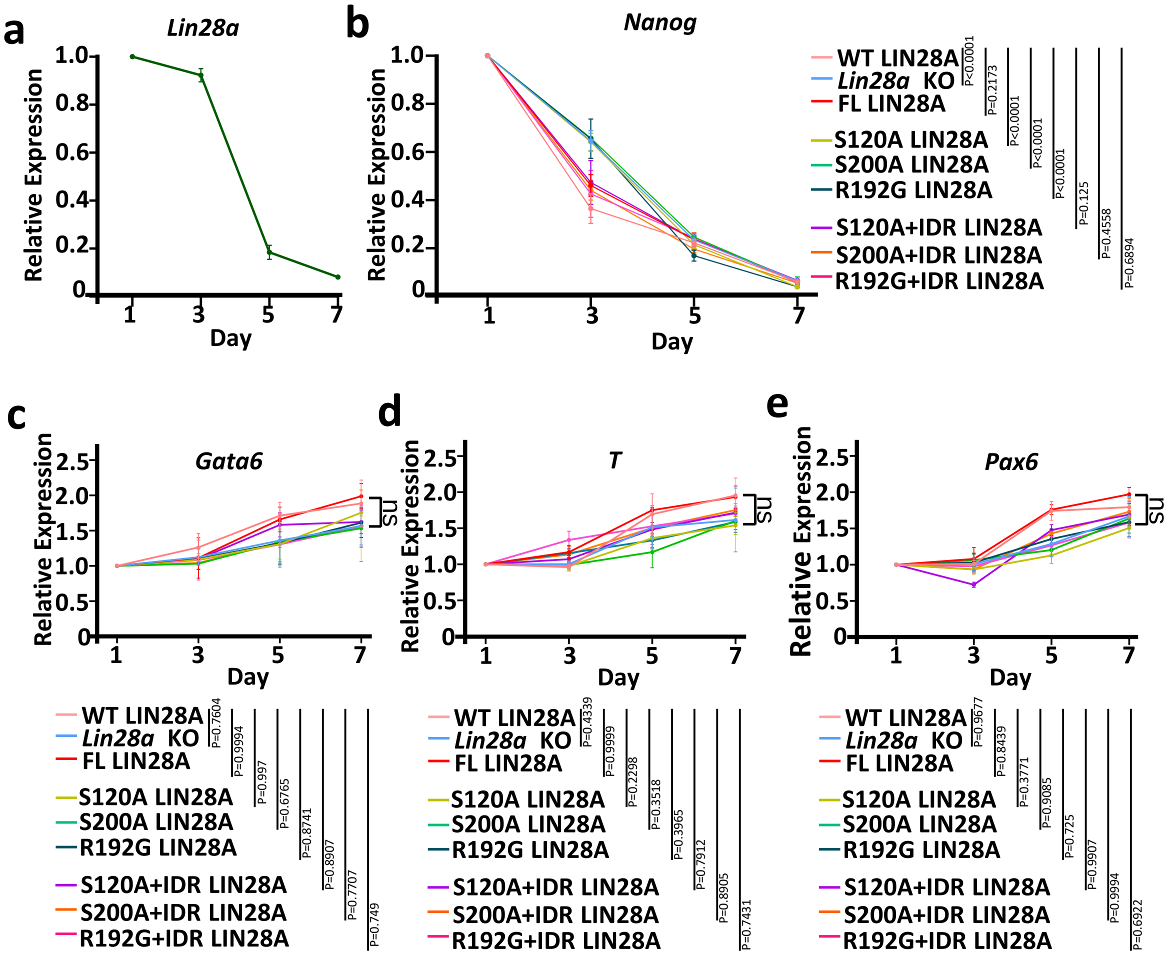

Supplementary Figure 8 The IDR mutant LIN28A did not affect the expression kinetics of genes in the three germ layers

a, qRT-PCR showing WT *Lin28a* gene expression after LIF withdrawal. n=3 biologically independent experiments. Data are presented as mean values +/− SEM. **b,** qRT-PCR showing pluripotency factor *Nanog* gene expression in WT, *Lin28a* KO, and *Lin28a* KO cells transduced with FL-LIN28A, S120A-LIN28A, S200A-LIN28A, R192G-LIN28A, and FUS protein's IDR fused LIN28A variants after LIF withdrawal. n=3 biologically independent experiments. Data are presented as mean values +/− SEM. Two-way ANOVA. **c,** qRT-PCR showing endoderm lineage gene *Gata6* expression in WT, *Lin28a* KO, and *Lin28a* KO cells transduced with FL-LIN28A, S120A-LIN28A, S200A-LIN28A, R192G-LIN28A, and FUS protein's IDR fused LIN28A variants after LIF withdrawal. n=3 biologically independent experiments. Data are presented as mean values +/− SEM. Two-way ANOVA. **d,** qRT-PCR showing mesoderm lineage gene *T* expression in WT, *Lin28a* KO, and *Lin28a* KO cells transduced with FL-LIN28A, S120A-LIN28A, S200A-LIN28A, R192G-LIN28A, and FUS protein's IDR fused LIN28A variants after LIF withdrawal. n=3 biologically independent experiments. Data are presented as mean values +/− SEM. Two-way ANOVA. **e,** qRT-PCR showing ectoderm lineage gene *Pax6* expression in WT, *Lin28a* KO, and *Lin28a* KO cells transduced with FL-LIN28A, S120A-LIN28A, S200A-LIN28A, R192G-LIN28A, and FUS protein's IDR fused LIN28A variants after LIF withdrawal. n=3 biologically independent experiments. Data are presented as mean values +/− SEM. Two-way ANOVA. Source data are provided as a Source Data file.

Supplementary Figure 9

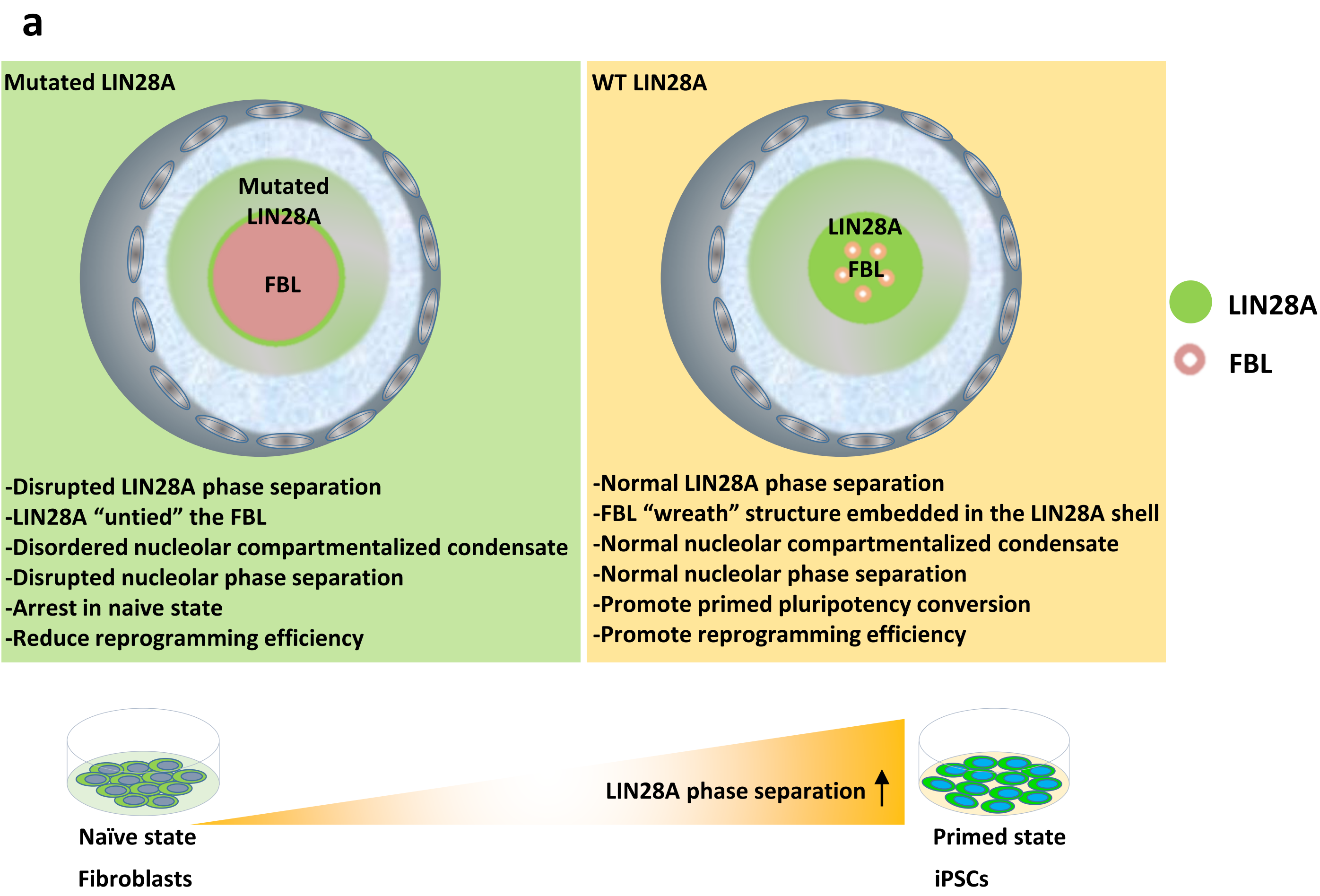

Supplementary Figure 9 A cartoon model for the role of LIN28A phase separation in maintaining nucleolar integrity and cell fate decision. a, In the WT ESCs, LIN28A phase separation maintains the normal liquid-liquid phase separation (LLPS) of the nucleolus. In contrast, in the LIN28A IDR mutation ESCs, abnormal LIN28A phase separation disrupted the natural liquid phase-separated structure of the nucleolus, which abolished the function of LIN28A in reprogramming and in the transition of naive-to-primed pluripotency states.

Table S1. The mouse primers for qPCR.

Gene name	Sequence
Nanog	F:5’-TCTTCCTGGTCCCCACAGTTT-3’<br>R:5’-GCAAGAATAGTTCTCGGGATGAA-3’
Klf4	F:5’-GTGCCCCGACTAACCGTTG-3’<br>R:5’-GTCGTTGAACTCCTCGGTCT-3’
Fgf5	F:5’-AAGTAGCGCGACGTTTTCTTC-3’<br>R:5’-CTGGAAACTGCTATGTTCCGAG-3’
Otx2	F:5’-TATCTAAAGCAACCGCCTTACG-3’<br>R:5’-AAGTCCATACCCGAAGTGGTC-3’
let-7g-5p	TGAGGTAGTAGTTTGTACAGTT
U6	F:5’-CGCTTCGGCAGCACATATAC-3’<br>R:5’-AAAATATGGAACGCTTCACGA-3’
Gata6	F:5’-GAACGTACCACCACCACCAT-3’<br>R:5’-CCATGTAGGGCGAGTAGGTC-3’
T	F:5’-CATGTACTCTTTCTTGCTGG-3’<br>R:5’-GGTCTCGGGAAAGCAGTGGC-3’
Pax6	F:5’-AAGGAGGGGGAGAGAACACC-3’<br>R:5’-TCTGAGCTTCATCCGAGTCTT-3’
Lin28a	F:5’-GGCATCTGTAAGTGGTTCAACG-3’<br>R:5’-CCCTCCTTGAGGCTTCGGA-3’
β-Actin	F:5’-TAGGCACCAGGGTGTGATGG-3’<br>R:5’-CATGGCTGGGGTGTTGAAGG-3’
Gapdh	F:5’-AGGTCGGTGTGAACGGATTTG-3’<br>R:5’-TGTAGACCATGTAGTTGAGGTCA-3’

Table S2. CRISPR/CAS9 sgRNA

Gene name	Sequence
Lin28a Knockout	5'-gccgctcgggccgcgtcagg-3'
Lin28a Knock-in	5'-cgggctcagcagacgaccat-3'
